# Diagnostic value of strand‐specific miRNA‐101‐3p and miRNA‐101‐5p for hepatocellular carcinoma and a bioinformatic analysis of their possible mechanism of action

**DOI:** 10.1002/2211-5463.12349

**Published:** 2017-12-20

**Authors:** Xia Yang, Yu‐Yan Pang, Rong‐Quan He, Peng Lin, Jie‐Mei Cen, Hong Yang, Jie Ma, Gang Chen

**Affiliations:** ^1^ Department of Pathology First Affiliated Hospital of Guangxi Medical University Nanning China; ^2^ Department of Medical Oncology First Affiliated Hospital of Guangxi Medical University Nanning China; ^3^ Department of Ultrasonography First Affiliated Hospital of Guangxi Medical University Nanning China

**Keywords:** bioinformatics, Gene Expression Omnibus, hepatocellular carcinoma, microRNA‐101‐3p, microRNA‐101‐5p, The Cancer Genome Atlas

## Abstract

There is accumulating evidence that miRNA might serve as potential diagnostic and prognostic markers for various types of cancer. Hepatocellular carcinoma (HCC) is the most common type of malignant lesion but the significance of miRNAs in HCC remains largely unknown. The present study aimed to establish the diagnostic value of miR‐101‐3p/5p in HCC and then further investigate the prospective molecular mechanism via a bioinformatic analysis. First, the miR‐101 expression profiles and parallel clinical parameters from 362 HCC patients and 50 adjacent non‐HCC tissue samples were downloaded from The Cancer Genome Atlas (TCGA). Second, we aggregated all miR‐101‐3p/5p expression profiles collected from published literature and the Gene Expression Omnibus and TCGA databases. Subsequently, target genes of miR‐101‐3p and miR‐101‐5p were predicted by using the miRWalk database and then overlapped with the differentially expressed genes of HCC identified by natural language processing. Finally, bioinformatic analyses were conducted with the overlapping genes. The level of miR‐101 was significantly lower in HCC tissues compared with adjacent non‐HCC tissues (*P* < 0.001), and the area under the curve of the low miR‐101 level for HCC diagnosis was 0.925 (*P* < 0.001). The pooled summary receiver operator characteristic (SROC) of miR‐101‐3p was 0.86, and the combined SROC curve of miR‐101‐5p was 0.80. Bioinformatic analysis showed that the target genes of both miR‐101‐3p and miR‐101‐5p are involved in several pathways that are associated with HCC. The hub genes for miR‐101‐3p and miR‐101‐5p were also found. Our results suggested that both miR‐101‐3p and miR‐101‐5p might be potential diagnostic markers in HCC, and that they exert their functions via targeting various prospective genes in the same pathways.

**Abbreviations**
AFP: alpha‐fetoprotein
AUC: area under the curve
BP: biological process
CC: cellular component
CI: confidence interval
CNKI: Chinese National Knowledge Infrastructure
DAVID: Database for Annotation, Visualization and Integrated Discovery
FN: false negative
FoxO: forkhead box O
FP: false positive
GEO: Gene Expression Omnibus
GO: Gene Ontology
HBV: hepatitis B virus
HCC: hepatocellular carcinoma
KEGG: Kyoto Encyclopedia of Genes and Genomes
MAPK: mitogen‐activated protein kinase
MF: molecular function
NLP: natural language processing
NLR: negative likelihood ratio
PI3K: phosphoinositide‐3‐kinase
PLR: positive likelihood ratio
PPI: protein–protein interaction
pre‐miRNA: precursor miRNA
ROC: receiver operating characteristic
SROC: summary receiver operator characteristic
TCGA: The Cancer Genome Atlas
TN: true negative
TP: true positive

According to Cancer Statistics, 2017 1, the incidence rates of liver cancer in the USA continue to increase rapidly (~ 3% per year in women and 4% per year in men), and the death rate rose by almost 3% per year from 2010 to 2014. In addition, the mortality rate is three times higher in men than in women. Since Asia is the area with the highest incidence rate of liver cancer, especially China, annual incidence and mortality are more than half of the global totals 2. Among the three histological types of liver malignancy, hepatocellular carcinoma (HCC) has become the leading cause of death from cancer. Since there have been no biomarkers or common surgical techniques for the early stage of HCC, the majority of patients with HCC are diagnosed late, which directly correlates with a poor outcome and low survival rate. As with other cancers, HCC development is a multistep process with abundant genetic and epigenetic mutations. A recent study confirmed that hepatocarcinogenesis can be caused by chronic hepatitis B virus (HBV) infection 3. Much effort towards the treatment of HBV‐infected HCC has been made in the past, but with only limited success. Thus, identifying novel biochemical markers for early HCC diagnosis is a matter of the utmost urgency.

miRNAs, ~ 20–22 nucleotides in length, are a class of small endogenous non‐coding RNA molecules. They post‐transcriptionally regulate mRNA expression through imperfect base paring with the 3′‐untranslated region of target genes. With comprehensive study, miRNAs have become known as the star molecules of cancer research. miRNAs in human cancers are involved in several pivotal biological processes (BP), including cancer proliferation, differentiation, progression and cell apoptosis 4, 5, 6. Although their functions remain elusive, up‐ and down‐regulation of miRNAs have been widely reported in all kinds of cancer tissues in comparison with expression in the corresponding normal tissues 7, 8. In particular, miRNAs have been found to be biomarkers for cancer clinical diagnosis, histological classification and prognosis 9, 10, 11, 12, 13.

Accumulating evidence has clearly demonstrated that the aberrant expression of miRNAs may further influence the expression of tumor oncogenes and suppressor genes, thereby leading to the occurrence of a tumor 14, 15, 16, 17. Theoretically, mature miRNA generation requires a series of enzyme reactions. First, primary miRNA transcripts are cleaved in the nucleus by the Drosha enzyme to liberate the precursor miRNA (pre‐miRNA) hairpin. Subsequently, the pre‐miRNA is exported to the cytoplasm and further processed by the enzyme Dicer to produce two mature miRNAs (miR‐5p and miR‐3p) 18, 19. Even though the two mature miRNAs are transcribed from the same pre‐miRNA, they may have different target genes and biological functions. A previous study 20 reported that the expression levels of the miR‐5p and miR‐3p mature sequences can be altered in different tissues.

Accumulating evidence 19, 21, 22, 23, 24, 25 has shown that miR‐101‐3p/‐5p is down‐regulated in multiple malignances, including HCC. For example, Hou *et al*. 26 explored miRNA expression profiling and revealed that miR‐101 (3p and 5p were not distinguished) expression in HCC tissues was lower than in healthy controls. Wei *et al*. 27 also showed that miR‐101 (3p and 5p were not distinguished) was down‐regulated in HBV‐associated HCC tissues and may have therapeutic potential in HCC. Additionally, the function of these miRNAs has also been investigated. Zhang *et al*. 28 revealed that enforced expression of miR‐101 (3p and 5p were not distinguished) by siRNA inhibited the cell proliferation and tumorigenicity of an HCC cell line *in vitro*. Sheng *et al*. 29 investigated how miR‐101‐3p regulated cell proliferation, cell cycle and apoptosis in HCC and found that overexpression of miR‐101‐3p caused an enhanced rate of apoptosis but no obvious change in the cell cycle. Besides, several oncogenes, such as *EZH2*,* FOS*,* COX‐2* and *SOX9*, have been found to be directly regulated by miR‐101‐3p/5p 30, 31, 32. Recently the potential of the miR‐101 family as diagnostic indicators has also caught the eye of researchers. He *et al*. 33 conducted a meta‐analysis that summarized miRNAs’ diagnostic value in HCC and found that miR‐101‐5p had great diagnostic value, though only three data sets were included and the results need to be further validated. Furthermore, in human, miR‐101 precursor transcripts are encoded with two genomic loci (miR‐101‐1 and miR‐101‐2). For two mature miRNAs, miR‐101‐3p is generated from the 3′ ends of the precursors, and miR‐101‐5p from the 5′ end of pre‐miR‐101‐1 (http://www.mirbase.org/). We speculated that miR‐101‐3p may also serve as a diagnostic marker for HCC. Since the seed region of miR‐101‐3p and miR‐101‐5p is unique, they are predicted to regulate unique targets. However, to the best of our knowledge, the comparative roles of miR‐101‐3p and miR‐101‐5p in HCC have not yet been fully studied.

The present study investigated miR‐101‐3p and miR‐101‐5p expression in HCC tissues compared with that in healthy controls. Published studies, Gene Expression Omnibus (GEO) microarray chips and The Cancer Genome Atlas (TCGA) data that included miR‐101‐3p or miR‐101‐5p expression information were collected together. Additionally, previous studies have mainly focused on a single gene 34, 35, 36, and studies have rarely focused on the function of coexpressed genes in cancers. For the purpose of obtaining a full understanding of the molecular mechanisms underlying HCC, comprehensive bioinformatics methods were used to investigate the function and pathways of target genes of miR‐101‐3p and miR‐101‐5p associated with HCC. In a word, the present study aimed to analyze the expression and mechanism of miR‐101‐3p and miR‐101‐5p in the initiation and development of HCC. This exploration will provide novel insights into HCC. A flowchart for the whole study designed is shown in Fig. 1.

**Figure 1:**
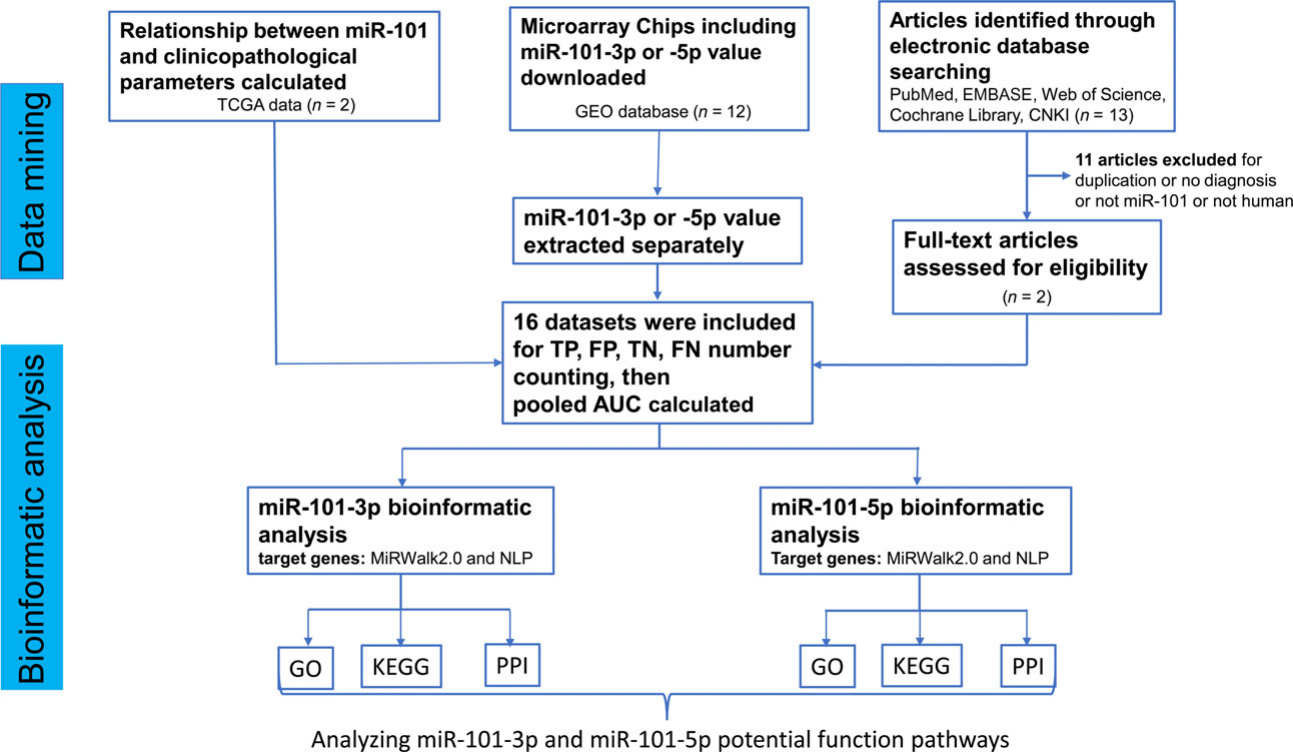
Flowchart of the study design.

## Material and methods

### The clinical role of miR‐101 based on the public database TCGA

To verify the difference in the miR‐101 expression levels between HCC and normal liver tissues, we downloaded relevant data from the public tumor database TCGA, in which samples from 362 HCC patients and 50 adjacent non‐HCC tissues were included. Additionally, miR‐101‐1 and miR‐101‐2 levels were both calculated because the relevant sample data were provided in TCGA. miR‐101‐1 and miR‐101‐2 are two precursor hairpin structures of miR‐101 miRNA that are located in the human genome on chromosome 1 (MI0000103) and 9 (MI0000739), respectively 37. Both of them are processed by the Dicer enzyme to form the mature miRNA. All of the available clinical parameters were analyzed by spss statistics 22.0 (IBM Corp., Armonk, NY, USA).

### Data mining

#### Search strategy and study selection

Comprehensive literature searches were conducted on electronic databases PubMed, EMBASE, Web of Science, the Cochrane Library, and Chinese National Knowledge Infrastructure (CNKI) up to 29 December 2016. No language limitations were imposed. Qualifying articles were screened by combining the following keywords: ‘miR‐101’ OR ‘miRNA‐101’ OR ‘miRNA‐101’ OR ‘miR101’ OR ‘miRNA101’ OR ‘miRNA 101’ OR ‘miR‐101‐5p’ OR ‘miRNA‐101‐5p’ OR ‘miRNA‐101‐5p’ OR'miR‐101‐3p’ OR ‘miRNA‐101‐3p’ OR ‘miRNA‐101‐3p’ AND malignan* OR cancer OR tumor OR neoplas* OR carcinoma AND hepatocellular OR liver OR hepatic OR HCC AND diagnos* OR receiver operating characteristic (ROC) OR specificity OR sensitivity OR DEGs OR DEMs OR ‘differentially expressed’. In addition, the reference lists were also manually searched to reduce article omission. The title and abstract of the obtained studies were scanned to exclude any clearly irrelevant publications. In addition to searching the literature, we also searched the GEO database for eligible microarrays with the following terms: malignan* OR cancer OR tumor OR neoplas* OR carcinoma AND hepatocellular OR liver OR hepatic OR HCC.

#### Criteria for inclusion and exclusion

Studies that met the following criteria were included: (a) investigated HCC; (b) measured the level of miR‐101, miR‐101‐3p or miR‐101‐5p in HCC tissue, plasma or serum; (c) included the diagnosis of HCC or the clinical parameters; and (d) reported true positives (TPs), false positives (FPs), false negatives (FNs), and true negatives (TNs) or sensitivity and specificity of miR‐101. In addition, (e) if the studies did not provide a fourfold contingency table, they were included if the original data were available; and (f) microarrays were included if they enrolled more than three patient samples and measured the miR‐101 profile for HCC.

Articles that met the following criteria were excluded: (a) studies without sufficient data, such as reviews or systematic reviews, (b) repeat reports, (c) studies conducted on cell lines or animals and (d) letters to the editor or conference abstracts.

#### Data synthesis and analysis

Studies that did not provide TPs, FPs, FNs and TNs but gave sensitivity and specificity or the original data were translated by medcalc 11.4.2.0 (MedCalc Software, Ostend, Belgium). To reduce inaccuracy in the relevant data extracted from the included studies, three independent researchers (XY, PL and JMC) performed the data extraction separately.

#### Statistical analysis

All statistical analyses were performed using spss statistics 20.0 or stata 12.0 (StataCorp, College Station, TX, USA). For the clinical parameter analysis, miR‐101 expression was represented as the mean ± standard deviation. The standards for assessing the area under the curve (AUC) in the ROC curve were as follows: 0.5–0.7 represented poor evidence for diagnosis, 0.7–0.9 represented moderate evidence for diagnosis and 0.9–1.0 represented high evidence for diagnosis. The correlation between miR‐101 expression and the clinicopathological parameters was investigated with Spearman's rank correlation. The significance of the difference between HCC and non‐cancerous liver tissues was studied using Student's *t* test. The significant differences among three groups were examined by one‐way ANOVA. For data mining, the pooled sensitivity, specificity, positive likelihood ratios (PLRs), negative likelihood ratios (NLRs), and diagnostic odds ratio with their corresponding 95% confidence intervals (CIs) were calculated with the bivariate regression model. Additionally, the summary receiver operator characteristic (SROC) curve with the area under the SROC curve was calculated 38. What is more, the *Q* test and the *I* ^2^ measure of inconsistency were used to quantify heterogeneity between studies 39. The possibility of publication bias was finally explored by Deeks’ funnel plot, and *P* values < 0.1 were considered significant.

### Bioinformatic analysis

#### *In silico* analysis of target genes of miR‐101

MiRWalk2.0 40 (http://zmf.umm.uni-heidelberg.de/apps/zmf/mirwalk2/), which combines 12 existing miRNA‐target prediction programs, was used to provide comprehensive potential targets for miR‐101‐3p and miR‐101‐5p. The genes identified by more than eight prediction software programs for miR‐101‐3p and more than six for miR‐101‐5p were selected to obtain more reliable targets.

#### Natural language processing

Natural language processing (NLP) is a novel computerized approach to analyze electronic free text to achieve ‘human‐like language processing’. With this approach, programmers create software to ‘read’ text and extract key pieces of information from clinician notes, procedure/radiology/pathology reports and laboratory results 41, 42. We performed a literature search in PubMed to obtain all related electronic records. The detailed process was described in our previous article 43, 44. Finally, 1800 genes that were differentially expressed in HCC were identified for further analysis.

#### Functional and signaling pathway analyses

A set of condition‐specific genes from the overlapping genes from the target prediction software and NLP further underwent functional and signaling pathway analyses on a public database platform, the Database for Annotation, Visualization and Integrated Discovery (DAVID; https://david.ncifcrf.gov/), which provides a functional interpretation of massive gene lists derived from genomic studies. The analyses included Gene Ontology (GO) function analysis (http://www.geneontology.org/) and Kyoto Encyclopedia of Genes and Genomes (KEGG; http://www.genome.jp/kegg/) analysis. The GO function analysis categorized selected genes into groups in accordance with three independent classification standards, BPs, cellular components (CCs), and molecular functions (MFs). The top 10 terms of each GO category and top 30 pathways of the KEGG pathways were visualized as GO maps and KEGG maps, separately, via cytoscape v3.4.0 (http://cytoscape.org/).

#### Protein–protein interaction network construction

Overlapping genes were inputted to the string v10.0 online tool (http://string-db.org/) to construct the protein–protein interaction (PPI) network. The direct (physical) and indirect (functional) associations of proteins were derived from four methods: (a) literature‐reported protein interactions, (b) high‐throughput experiments, (c) genome analysis and prediction and (d) coexpression studies. By scrutinizing the connectivity degrees of the nodes in the PPI networks, we determined the hub genes. A node with a high degree of connectivity is perceived as a hub node.

## Results

### Clinicopathological significance of miR‐101‐1/miR‐101‐2 in HCC tissues

**Figure 2:**
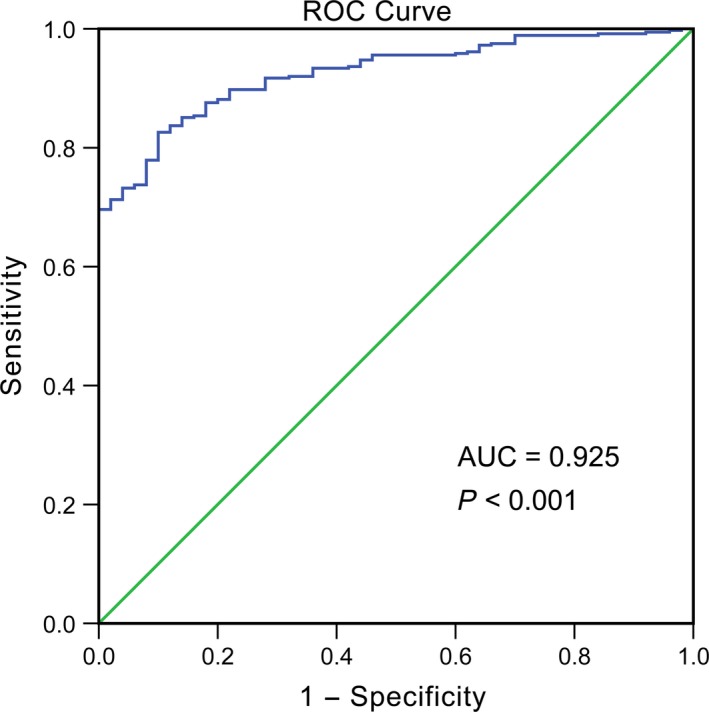
MiR‐101 expression profiles for the diagnosis of HCC. The AUC of the low miR‐101 level for HCC diagnosis was 0.925 (95% CI: 0.896–0.953, *P* < 0.001).

The relationship between miR‐101‐1/miR‐101‐2 and clinicopathological parameters in HCC was mined from TCGA, as shown in Tables 1 and 2. Data profiling revealed that when compared with the expression in para‐non‐cancerous normal tissues (16.93 ± 0.62), miR‐101‐1 expression was significantly reduced in HCC tissues (15.25 ± 1.05, *P* < 0.001). In addition, the AUC of the low miR‐101‐1 level for HCC diagnosis was 0.925 (95% CI: 0.896–0.953, *P* < 0.001, Fig. 2) with a cut‐off value of 16.17 (82.6% sensitivity and 90.0% specificity). Similar results were also obtained for miR‐101‐2 (15.27 ± 1.05 in HCC and 16.94 ± 0.61 in para‐non‐cancerous liver tissues, *P* < 0.001). Additionally, the altered expressions of miR‐101‐1 and miRNA‐101‐2 were both associated with pathological stage, pathological T stage and histological stage. Compared with the expression in advanced stage (III and IV) HCC patients, the relative expression of miR‐101 in early stage patients was notably increased (I and II, *P* < 0.05), and the Spearman correlation test confirmed that the correlations between miR‐101 and the pathological stage, pathological T stage and histological stage were *r* = −0.17, *P* = 0.001; *r* = −0.17, *P* = 0.001 and *r* = −0.18, *P* < 0.001, respectively.

**Table 1:** Relationship between miR‐101‐1 and clinicopathological parameters in HCC (TCGA data)

Clinicopathological feature	miR‐101‐1 relative expression				Correlation analysis	
	*n*	Mean ± SD	*t*	*P*	*r*	*P*
Tissue						
HCC	362	15.25 ± 1.05	−16.198	0.000	0.480	0.000
Normal	50	16.93 ± 0.62				
Gender						
Male	247	15.23 ± 1.03	−0.542	0.588	0.015	0.774
Female	115	15.29 ± 1.09				
Age						
< 50 years	68	15.06 ± 1.13	−1.708	0.089	0.031	0.559
≥ 50 years	290	15.30 ± 1.03				
HBV						
−	255	15.20 ± 1.08	1.347	0.179	0.079	0.133
+	106	15.37 ± 0.98				
HCV						
−	308	15.22 ± 1.05	1.407	0.160	0.054	0.309
+	54	15.44 ± 1.01				
Pathological stage						
Stage I–II	250	15.34 ± 1.01	3.276	0.001	−0.174	0.001
Stage III–IV	88	14.92 ± 1.16				
Pathological T						
T1–T2	268	15.33 ± 1.01	2.867	0.004	−0.173	0.001
T3–T4	92	14.97 ± 1.12				
Histological grade						
GI–II	223	15.40 ± 1.05	3.467	0.001	−0.184	0.000
GIII–IV	135	15.00 ± 1.02				

**Table 2:** Relationship between miR‐101‐2 and clinicopathological parameters in HCC (TCGA data)

Clinicopathological feature	miR‐101‐2 relative expression				Correlation analysis	
	*n*	Mean ± SD	*t*	*P*	*r*	*P*
Tissue						
HCC	362	15.27 ± 1.05	−16.256	0.000	0.481	0.000
Normal	50	16.94 ± 0.61				
Gender						
Male	247	15.25 ± 1.03	−0.533	0.594	0.016	0.760
Female	115	15.31 ± 1.09				
Age						
< 50 years	68	15.08 ± 1.13	−1.709	0.088	0.032	0.550
≥ 50 years	290	15.32 ± 1.03				
HBV						
−	255	15.22 ± 1.07	1.347	0.179	0.079	0.133
+	106	15.39 ± 0.98				
HCV						
−	308	15.24 ± 1.05	1.425	0.155	0.054	0.309
+	54	15.46 ± 1.01				
Pathological stage						
Stage I–II	250	15.36 ± 1.01	3.309	0.001	−0.174	0.001
Stage III–IV	88	14.93 ± 1.15				
Pathological T						
T1–T2	268	15.35 ± 1.00	2.904	0.004	−0.174	0.001
T3–T4	92	14.99 ± 1.12				
Histological grade						
GI–II	223	15.41 ± 1.04	3.452	0.001	−0.184	0.000
GIII–IV	135	15.03 ± 1.02				

### Diagnostic value of miR‐101‐3p and miR‐101‐5p

#### Study selection

Through the literature search, 341 relevant articles were identified, 339 of which were excluded for being case reports, reviews, letters, repeat publications and studies not specifically pertaining to miR‐101‐3p/5p. The two remaining publications were examined by three researchers and ultimately included. Moreover, GEO microarrays that detected miR‐101‐3p and/or miR‐101‐5p were identified for further analysis and were combined after assessment. Finally, 12 datasets including 315 HCC and 330 normal control samples were downloaded from the GEO database to calculate the miR‐101‐3p diagnostic value (GSE39678, GSE21279, GSE67882, GSE65708, GSE12717, GSE10694, GSE22058, GSE21362, GSE40744, GSE41874, GSE54751 and GSE57555); five datasets including 308 HCC and 114 normal control samples were downloaded from the GEO database to calculate the miR‐101‐5p diagnostic value (GSE74618, GSE21362, GSE40744, GSE41874 and GSE57555). In addition, the precursors of miR‐101 identified from TCGA were also considered.

#### Heterogeneity analysis

The analysis of heterogeneity is widely used to evaluate the accuracy of statistical pooling from multiple studies 45. Since heterogeneities may come from a threshold effect and a non‐threshold effect, the threshold effect was first explored by the Spearman test to calculate the heterogeneity of miR‐101‐3p/5p among the included studies. In other words, the correlation coefficient and *P* value between the logit of sensitivity and logit of 1 – specificity were calculated. As a result, the Spearman correlation coefficients for miR‐101‐3p and miR‐101‐5p were 0.386 (*P* = 0.215) and −0.059 (*P* = 0.912), respectively, indicating that heterogeneity from the threshold effect was not found. However, the *I* ^2^ values in the forest plots of sensitivity and specificity (more than 50%) revealed that we cannot ignore the non‐threshold effect from the included studies.

#### Diagnostic accuracy of miR‐101‐3p and miR‐101‐5p in HCC

**Figure 3:**
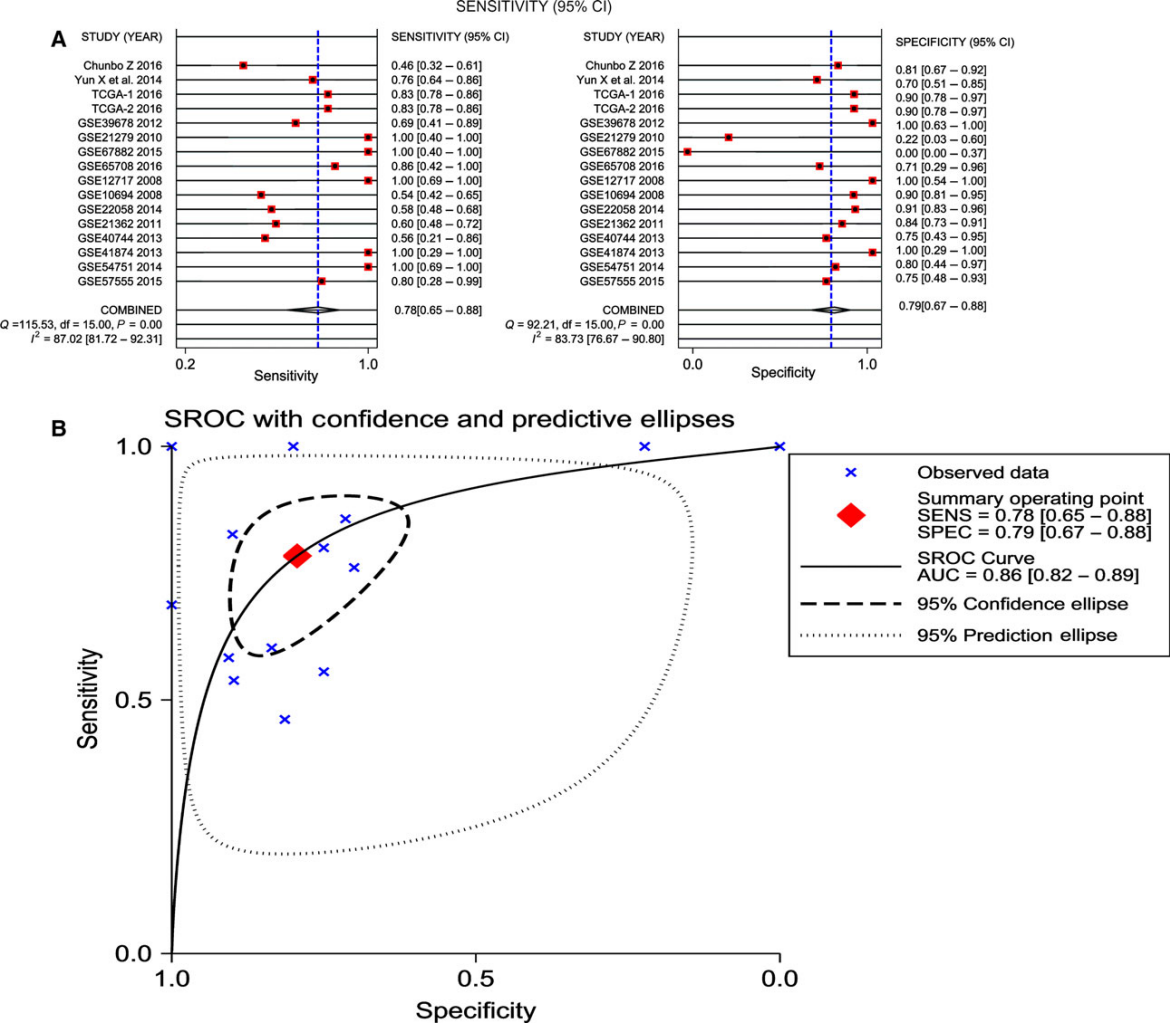
Diagnostic accuracy of miR‐101‐3p in HCC. (A) Sensitivity (SENS) and specificity (SPEC) with corresponding heterogeneity statistics. (B) SROC curves for miR‐101‐3p with CI in the diagnosis of HCC.

**Figure 4:**
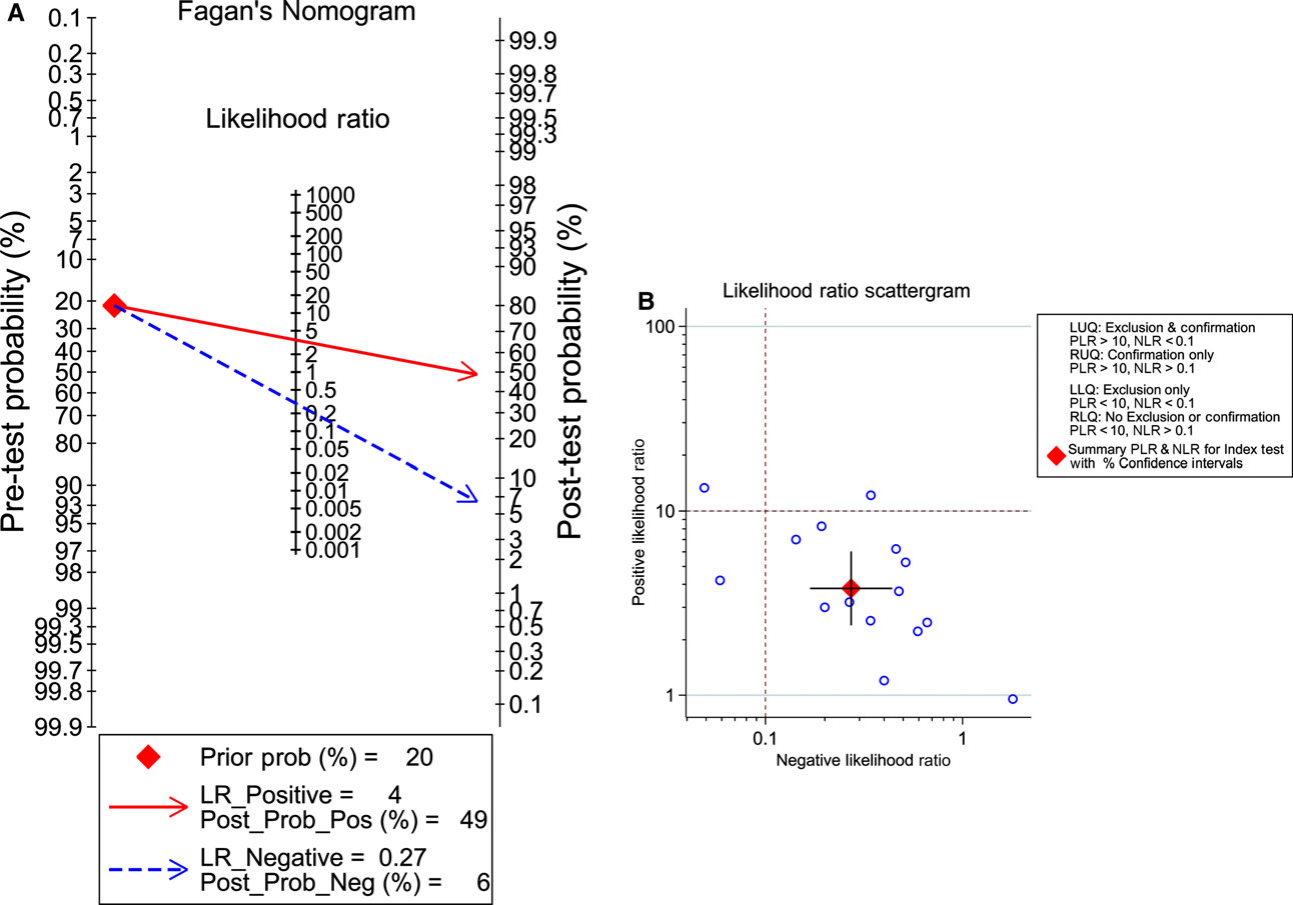
Fagan diagram and likelihood matrix for miR‐101‐3p to diagnose cancer or to eliminate the diagnosis of cancer. (A) Pre‐test probability of the miR‐101‐3p assay in HCC detection. (B) Likelihood matrix showing individual (circles) and pooled (diamond) values of PLRs combined with NLRs. LLQ, left lower quadrant; LUQ, left upper quadrant; RLQ, right lower quadrant; RUQ, right upper quadrant.

Evident heterogeneity for pooled sensitivity and specificity of miR‐101‐3p was seen in the collected data (*I* ^2^ = 87.02% and 83.73%, respectively, *P* < 0.05); thus, a random effects model was finally selected, based on which the pooled sensitivity and specificity of miR‐101‐3p was 78.0% (95% CI: 65.0–88.0%) and 79.0% (95% CI: 67.0–88.0%), respectively (Fig. 3A). In addition, the summary SROC of miR‐101‐3p was 0.86 (95% CI: 0.82–0.89; Fig. 3B). In addition, the PLR and NLR for HCC diagnosis were 3.803 (95% CI: 2.398–6.033) and 0.272 (95% CI: 0.169–0.439), respectively. Furthermore, the pre‐test probability was 20 for miR‐101‐3p, and the corresponding positive and negative post‐test probabilities of miR‐101‐3p were 49 and 6, respectively, suggesting that the power of miR‐101‐3p to diagnose real patients as HCC was 3.8 times the normal control (Fig. 4A). In addition, the likelihood ratio scattergram disclosed that the summary point of the PLR together with the NLR lies in the right lower quadrant (PLR < 10, NLR > 0.1; Fig. 4B).

**Figure 5:**
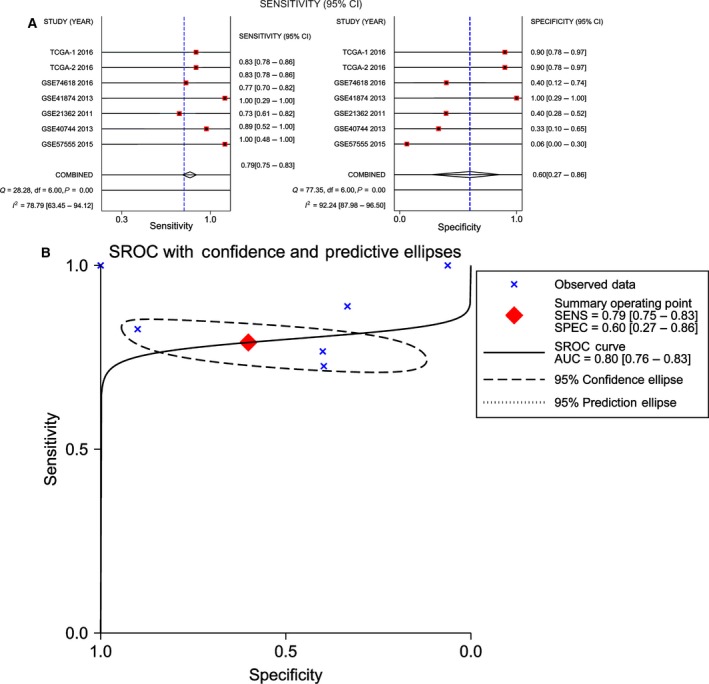
Diagnostic accuracy of miR‐101‐5p in HCC. (A) Sensitivity (SENS) and specificity (SPEC) with corresponding heterogeneity statistics. (B) SROC curves for miR‐101‐5p with CI in the diagnosis of HCC.

**Figure 6:**
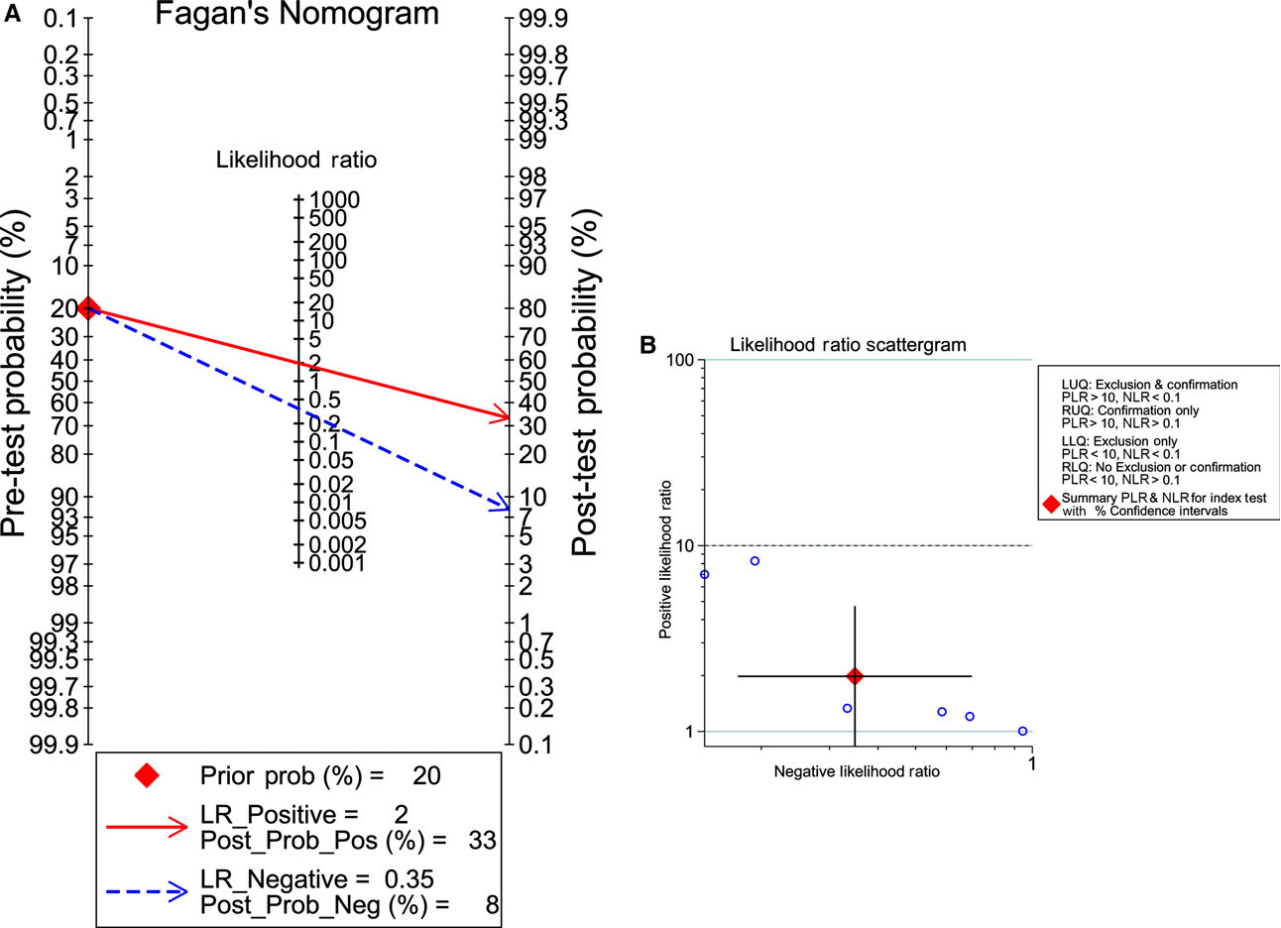
Fagan diagram and likelihood matrix for miR‐101‐5p to diagnose cancer or to eliminate the diagnosis of cancer. (A) Pre‐test probability of the miR‐101‐5p assay in HCC detection. (B) Likelihood matrix showing individual (circles) and pooled (diamond) values of PLRs combined with NLRs. LLQ, left lower quadrant; LUQ, left upper quadrant; RLQ, right lower quadrant; RUQ, right upper quadrant.

With regard to miR‐101‐5p, a random effects model was also selected for further analysis (*I* ^2^ = 78.79% and 92.24%, for sensitivity and specificity, respectively, *P* < 0.01). The overall sensitivity and specificity were 79.0% (95% CI: 75.0–83.0%) and 60.0% (95% CI: 27.0–86.0%), respectively (Fig. 5A). The calculated AUC of the summary SROC was 0.80 (95% CI: 0.76–0.83; Fig. 5B). Additionally, the PLR was 1.981 (95% CI: 0.831–4.721), and the NLR was 0.349 (95% CI: 0.174–0.698); the pre‐test probability of miR‐101‐5p was 20, and the corresponding positive and negative post‐test probability of miR‐101‐5p was 33 and 8, respectively, suggesting that the power of miR‐101‐5p to diagnose real patients as HCC was 1.98 times the normal control (Fig. 6A). In addition, the summary point of the PLR combined with the NLR also lies in the right lower quadrant (PLR < 10, NLR > 0.1), which was consistent with the Fagan's nomogram result (Fig. 6B).

#### Publication bias

**Figure 7:**
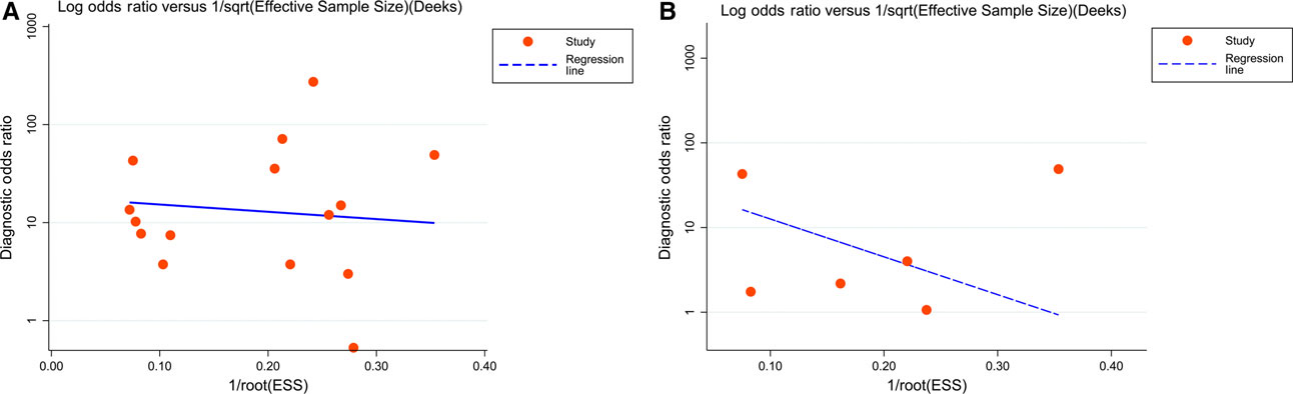
The Deeks’ test that assesses potential publication bias in the miR‐101 assay. (A) Potential publication bias assessment of miR‐101‐3p. (B) Potential publication bias assessment of miR‐101‐5p.

Publication bias was conducted by using the Deeks’ funnel plot asymmetry test. According to the results, the funnel plots that represented every study were almost symmetric, suggesting that publication bias from the studies included was absent in our study. The obtained *P*‐values of 0.718 and 0.447 for miR‐101‐3p and miR‐101‐5p, respectively, also revealed the absence of publication bias (Fig. 7).

### Bioinformatic analysis

To improve understanding of the function of miR‐101, the potential target genes of miR‐101‐3p and miR‐101‐5p in HCC were identified separately. Based on the prediction software and NLP, 73 target genes corresponding to miR‐101‐3p and 90 target genes corresponding to miR‐101‐5p were obtained. Subsequently, bioinformatic analyses were conducted to investigate the function and pathways of target genes of miR‐101 associated with HCC. All of the target genes were inputted into DAVID for bioinformatic analysis.

#### KEGG pathway enrichment analysis

**Figure 8:**
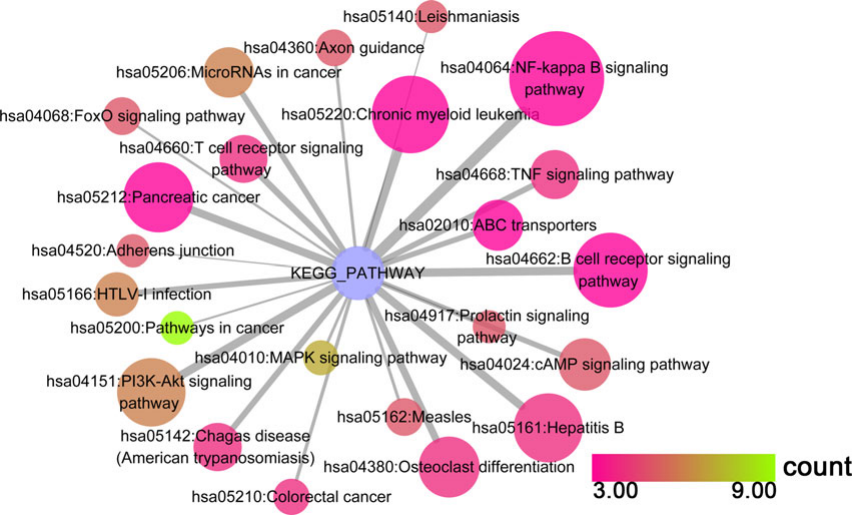
The KEGG pathway analysis of miR‐101‐3p predicted target genes in HCC. Pathway analyses were performed to identify significantly enriched pathways by using cytoscape v3.4.0. The top 30 pathways are displayed; the map node size represents the *P* value of targets, low values are indicated by large nodes, and the node color represents the gene count number with low values indicated by pink.

**Figure 9:**
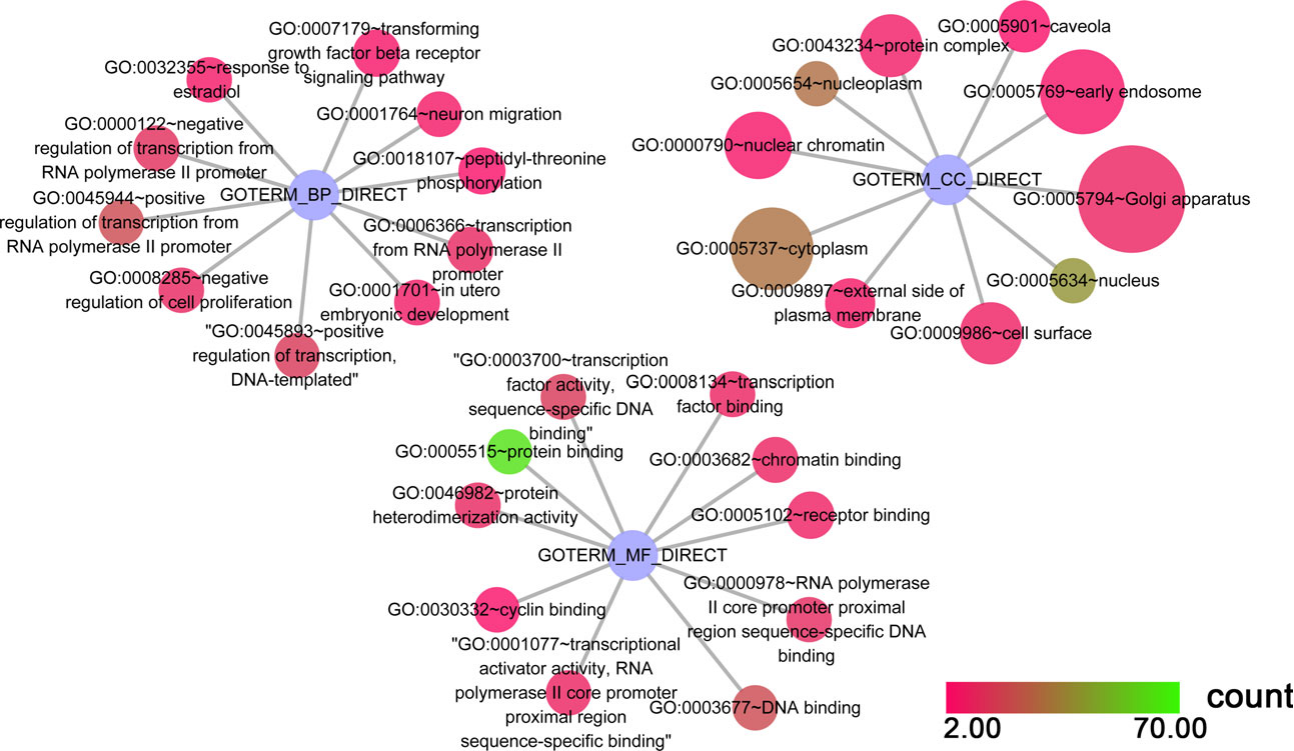
The KEGG pathway analysis of miR‐101‐5p predicted target genes in HCC. Pathway analyses were performed to identify significantly enriched pathways by using cytoscape v. 3.4.0. The top 30 pathways are displayed; the map node size represents the *P* value of targets, low values are indicated by large nodes, and the node color represents the gene count number with low values indicated by pink.

Our study revealed that 23 KEGG pathways corresponding to miR‐101‐3p were enriched, from which the top five pathways in which target genes were enriched were (a) the adherens junction pathway (hsa04520: *P* = 8.53 × 10^−4^), (b) the leishmaniasis pathway (hsa05140: *P* = 8.53 × 10^−4^), (c) the prolactin signaling pathway (hsa04917: *P* = 8.53 × 10^−4^), (d) pathways in cancer (hsa05200: *P* = 0.002) and (e) the mitogen‐activated protein kinase (MAPK) signaling pathway (hsa04010: *P* = 0.004). For miR‐101‐5p, 90 KEGG pathways were enriched, and the top pathways were (a) pathways in cancer (hsa05200: *P* = 7.08 × 10^−16^), (b) the forkhead box O (FoxO) signaling pathway (hsa04068: *P* = 5.59 × 10^−14^), (c) hepatitis B (hsa05161: *P* = 2.82 × 10^−12^), (d) the phosphoinositide‐3‐kinase (PI3K)–Akt signaling pathway (hsa04151: *P* = 7.91 × 10^−11^) and (e) the prolactin signaling pathway (hsa04917: *P* = 2.43 × 10^−8^). The top 30 pathways associated with miR‐101‐3p and miR‐101‐5p are shown in Figs 8 and 9, respectively, and all of the pathways are displayed in Table 3.

**Table 3:** KEGG functional annotation for most significantly related targets of miR‐101

KEGG ID	Term	Gene no.	*P*	Genes
MiR‐101‐3p				
hsa04520	Adherens junction	5	8.53 × 10^−4^	MAP3K7, MAPK1, TGFBR1, NLK, SSX2IP
hsa05140	Leishmaniasis	5	8.53 × 10^−4^	MAP3K7, MAPK1, FOS, PTGS2, JAK2
hsa04917	Prolactin signaling pathway	5	8.53 × 10^−4^	MAPK1, FOS, SOCS2, GSK3B, JAK2
hsa05200	Pathways in cancer	9	2.18 × 10^−3^	CEBPA, MAPK1, FOS, PTGS2, TGFBR1, GSK3B, PTCH1, CDK6, CXCL12
hsa04010	MAPK signaling pathway	7	4.09 × 10^−3^	MAP3K7, MAPK1, FOS, DUSP1, TGFBR1, NLK, SRF
hsa05210	Colorectal cancer	4	6.16 × 10^−3^	MAPK1, FOS, TGFBR1, GSK3B
hsa04360	Axon guidance	5	7.10 × 10^−3^	MAPK1, EPHA7, NRP1, GSK3B, CXCL12
hsa05162	Measles	5	8.34 × 10^−3^	MAP3K7, GSK3B, IL13, CDK6, JAK2
hsa04068	FoxO signaling pathway	5	8.56 × 10^−3^	MAPK1, TGFBR1, NLK, CCNG2, BCL2L11
hsa05166	HTLV‐I infection	6	1.86 × 10^−2^	FOS, NRP1, ETS1, TGFBR1, GSK3B, SRF
MiR‐101‐5p				
hsa05200	Pathways in cancer	35	7.08 × 10^−16^	XIAP, PTGS2, FOXO1, MMP1, TPM3, CCNE2, IGF1R, KRAS, CDKN2B, BCL2, SOS1, ITGAV, MYC, AKT3, PRKCA, BMP4, IL6, RALBP1, TGFBR1, CREBBP, *et al*.
hsa04068	FoxO signaling pathway	21	5.59 × 10^−14^	IL6, SGK3, TGFBR1, CREBBP, SMAD4, SMAD3, FOXO1, SMAD2, MAPK10, IL7R, CCNG2, BCL2L11, ATM, IGF1R, NRAS, MAPK1, KRAS, CDKN2B, CCND2, SOS1, AKT3
hsa05161	Hepatitis B	20	2.82 × 10^−12^	PRKCA, IL6, YWHAZ, TGFBR1, CREBBP, MAP2K4, CYCS, SMAD4, CDK6, MAPK10, STAT2, CCNE2, NRAS, MAPK1, KRAS, DDX3X, BCL2, NFATC2, MYC, AKT3
hsa04151	PI3K–Akt signaling pathway	27	7.91 × 10^−11^	YWHAZ, RPS6KB1, IL7R, CCNE2, IGF1R, KRAS, SOS1, ITGAV, BCL2, ANGPT2, MYC, AKT3, GHR, PRKCA, IL6, FLT1, SGK3, MET, CDK6, BCL2L11, *et al*.
hsa04917	Prolactin signaling pathway	12	2.43 × 10^−8^	MAPK1, NRAS, KRAS, TNFRSF11A, SOCS2, PRLR, CCND2, SOS1, ESR1, ESR2, MAPK10, AKT3
hsa04520	Adherens junction	12	2.43 × 10^−8^	MAP3K7, MAPK1, IGF1R, TGFBR1, CREBBP, MET, SMAD4, SMAD3, SMAD2, WASL, YES1, CTNNA1
hsa05220	Chronic myeloid leukemia	12	2.83 × 10^−8^	MAPK1, NRAS, KRAS, CRKL, HDAC2, TGFBR1, SOS1, SMAD4, CDK6, MYC, AKT3, PTPN11
hsa05205	Proteoglycans in cancer	18	3.80 × 10^−8^	PRKCA, ERBB4, MET, ESR1, RPS6KB1, SDC2, FZD7, PTPN11, IGF1R, NRAS, MAPK1, KRAS, ITGAV, SOS1, MYC, FRS2, AKT3, TWIST1
hsa05210	Colorectal cancer	11	7.32 × 10^−8^	MAPK1, KRAS, TGFBR1, BCL2, CYCS, SMAD4, SMAD3, SMAD2, MAPK10, MYC, AKT3
hsa04630	Jak–STAT signaling pathway	15	1.37 × 10^−7^	IL6, SOCS2, IL6ST, LEPR, CREBBP, IL7R, STAT2, PTPN11, LEP, PRLR, CCND2, SOS1, MYC, AKT3, GHR

#### GO enrichment analysis

**Figure 10:**
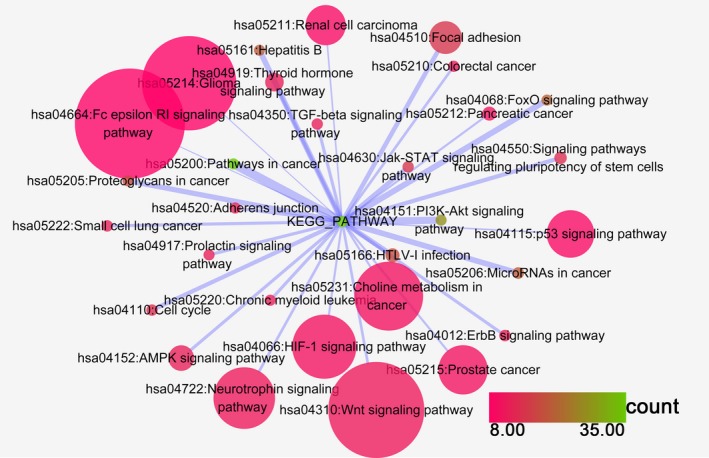
GO functional analysis of miR‐101‐3p in HCC. Top 10 terms of each category are displayed, and every node represents different BP terms; the map node size represents the *P* value of targets, low values are indicated by large nodes, and the node color represents the gene count number with low values indicated by pink.

**Figure 11:**
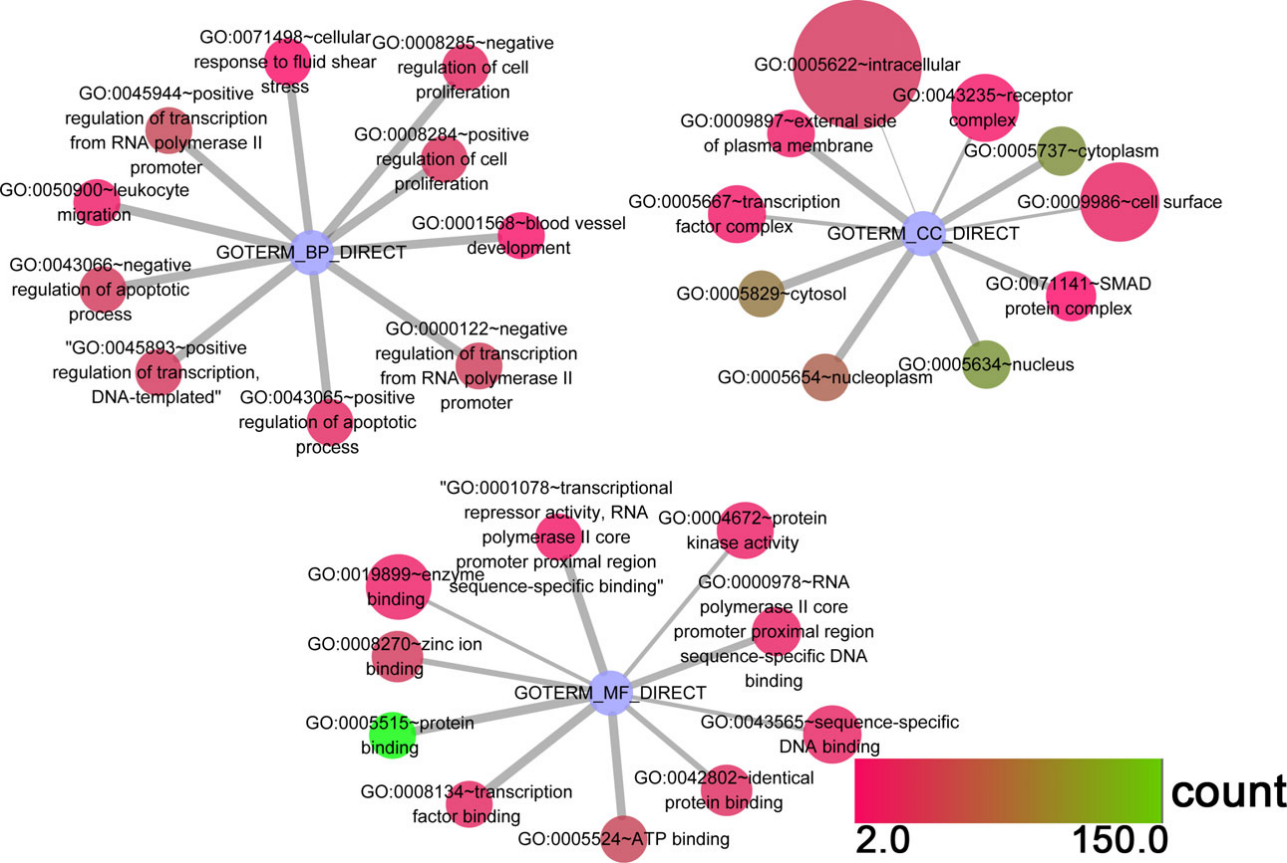
GO functional analysis of miR‐101‐5p in HCC. Top 10 terms of each category are displayed, and every node represents different BP terms; the map node size represents the *P* value of targets, low values are indicated by large nodes, and the node color represents the gene count number with low values indicated by pink.

As shown in Tables 4 and 5, the GO enrichment was composed of the BP, CC and MF categories. In the BP category for miR‐101‐3p, we can observe that the 73 target genes were mainly enriched in (a) positive regulation of transcription from the RNA polymeraseIIpromoter (GO: 0045944, *P* = 1.90 × 10^−8^), (b) positive regulation of transcription, DNA‐templated (GO: 0045893, *P* = 3.19 × 10^−8^) and (c) response to estradiol (GO: 0032355, *P* = 4.40 × 10^−5^). In addition, in the CC category, (a) nucleoplasm (GO: 0005654, *P* = 2.50 × 10^−7^), (b) nucleus (GO: 0005634, *P* = 1.24 × 10^−5^) and (c) external side of plasma membrane (GO: 0009897, *P* = 0.002) remained the top three enriched items. With regard to MF, the top ranking three items were protein binding (GO: 0005515, *P* = 1.67 × 10^−9^), RNA polymerase II core promoter proximal region sequence‐specific DNA binding (GO: 0000978, *P* = 2.20 × 10^−6^) and transcriptional activator activity, RNA polymerase II core promoter proximal region sequence‐specific binding (GO: 0001077, *P* = 6.46 × 10^−6^). In the same way, the top three enriched pathways of the 119 target genes of miR‐101‐5p in the BP category were (a) negative regulation of the apoptotic process (GO: 0043066, *P* = 2.01 × 10^−13^), (b) positive regulation of transcription, DNA‐templated, negative regulation of transcription from the RNA polymerase II promoter (GO: 0045893, *P* = 2.34 × 10^−11^) and (c) positive regulation of transcription from the RNA polymerase II promoter (GO: 0045944, *P* = 5.43 × 10^−11^). For the CC category, (a) cytosol (GO: 0005829, *P* = 3.59 × 10^−8^), (b) nucleoplasm (GO: 0005654, *P* = 1.23 × 10^−5^) and (c) external side of the plasma membrane (GO: 0009897, *P* = 1.50 × 10^−5^) were the most enriched terms. In addition, in the MF category, the utmost significant three items were (a) protein binding (GO: 0005515, *P* = 5.59 × 10^−14^), (b) transcription factor binding (GO: 0008131, *P* = 1.69 × 10^−8^) and (c) transcriptional repressor activity, RNA polymerase II core promoter proximal region sequence‐specific binding (GO: 0001078. *P* = 3.79 × 10^−6^). All of the GO enrichment items are visualized in the GO network (Figs 10 and 11).

**Table 4:** GO functional annotation for most significantly related targets of miR‐101‐3p

GO ID	Term	Gene no.	*P*	Genes
BP				
GO: 0045944	Positive regulation of transcription from RNA polymerase II promoter	20	1.90 × 10^−8^	CEBPA, SOX6, ZEB1, ZIC1, TET2, SOX9, PROX1, SRF, MYCN, PGR, MEF2D, FOS, ETS1, ZNF148, GSK3B, ASH1L, NEUROD1, TCF4, BCL9, SMARCA4
GO: 0045893	Positive regulation of transcription, DNA‐templated	15	3.19 × 10^−8^	KLF6, RSF1, TGFBR1, ARID1A, SOX9, ZIC1, PROX1, MYCN, MAPK1, FOS, ETS1, NEUROD1, PTCH1, TCF4, SMARCA4
GO: 0032355	Response to estradiol	6	4.40 × 10^−5^	DUSP1, SOCS2, PTGS2, ETS1, EZH2, PTCH1
GO: 0008285	Negative regulation of cell proliferation	10	4.72 × 10^−5^	CEBPA, PTGS2, ETS1, CDK6, JAK2, ZEB1, ARID2, PROX1, SRF, CDH5
GO: 0001764	Neuron migration	6	8.71 × 10^−5^	NRP1, GJA1, PAFAH1B1, TOP2B, CXCL12, SRF
GO: 0001701	*In utero* embryonic development	7	1.49 × 10^−4^	TGFBR1, MYO1E, GJA1, PTCH1, SOX6, SRF, BCL2L11
GO: 0000122	Negative regulation of transcription from RNA polymerase II promoter	12	2.32 × 10^−4^	CUL3, CEBPA, JDP2, ZNF148, EZH2, PTCH1, ARID1A, SOX6, ZEB1, TCF4, PROX1, SMARCA4
GO: 0006366	Transcription from RNA polymerase II promoter	10	3.32 × 10^−4^	CEBPA, MEF2D, FOS, ZNF148, ETS1, ASH1L, NEUROD1, ZIC1, SOX9, SRF
GO: 0018107	Peptidyl‐threonine phosphorylation	4	5.73 × 10^−4^	MAPK1, TGFBR1, GSK3B, NLK
GO: 0007179	Transforming growth factor beta receptor signaling pathway	5	6.53 × 10^−4^	MAP3K7, FOS, TGFBR1, NLK, CDH5
CC				
GO: 0005654	Nucleoplasm	30	2.50 × 10^−7^	ING3, RSF1, XPO5, XPO4, EZH2, ZEB1, SOX6, ZIC1, SOX9, SRF, ARID2, LARP1, CUL3, PGR, FOS, FBXW7, ZNF148, TOP2B, BCL9, NLK, *et al*.
GO: 0005634	Nucleus	40	1.24 × 10^−5^	ING3, JDP2, RSF1, PTGS2, XPO5, EZH2, SOX6, ZEB1, SOX9, ZIC1, TIMP3, SRF, MAP3K7, CUL3, PGR, FOS, MSI1, SSX2IP, TOP2B, TCF4, *et al*.
GO: 0009897	External side of plasma membrane	6	1.54 × 10^−3^	CLCN3, FGA, IL13, ABCA1, CXCL12, CDH5
GO: 0005901	Caveola	4	2.17 × 10^−3^	MAPK1, PTGS2, PTCH1, JAK2
GO: 0009986	Cell surface	8	5.50 × 10^−3^	CLCN3, NRP1, FGA, TGFBR1, CFTR, SPARC, CDH5, SLC7A11
GO: 0043234	Protein complex	7	5.73 × 10^−3^	MAPK1, FBXW7, PTGS2, CFTR, SSX2IP, SOX9, SMARCA4
GO: 0000790	Nuclear chromatin	5	7.16 × 10^−3^	EZH2, ARID1A, TCF4, SRF, SMARCA4
GO: 0005737	Cytoplasm	31	1.21 × 10^−2^	ING3, PTGS2, XPO5, XPO4, EZH2, IL13, ZEB1, ZIC1, CCNG2, SRF, LIN28B, LARP1, MAP3K7, FBXW7, MSI1, TOP2B, ZMYM2, SOCS2, MYO1E, CFTR, *et al*.
GO: 0005769	Early endosome	5	1.28 × 10^−2^	MAPK1, CLCN3, NRP1, GJA1, CFTR
GO: 0005794	Golgi apparatus	9	2.02 × 10^−2^	CUL3, MAPK1, CLCN3, ZNF148, ASH1L, GJA1, PTCH1, ABCA1, BCL9
MF				
GO: 0005515	Protein binding	62	1.67 × 10^−9^	JDP2, NRP1, RSF1, PTGS2, XPO5, XPO4, EZH2, IL13, GJA1, ZEB1, LARP1, MAP3K7, PGR, CUL3, FOS, ZNF148, SOCS2, CFTR, ARID1A, CDK6, *et al*.
GO: 0000978	RNA polymerase II core promoter proximal region sequence‐specific DNA binding	11	2.20 × 10^−6^	PGR, CEBPA, MEF2D, FOS, JDP2, ZNF148, NEUROD1, TCF4, ZIC1, SRF, SMARCA4
GO: 0001077	Transcriptional activator activity, RNA polymerase II core promoter proximal region sequence‐specific binding	9	6.46 × 10^−6^	PGR, CEBPA, MEF2D, FOS, NEUROD1, TCF4, ZIC1, SOX9, SRF
GO: 0008134	Transcription factor binding	9	2.47 × 10^−5^	CEBPA, MAPK1, FOS, ETS1, NLK, NEUROD1, ZEB1, SRF, SMARCA4
GO: 0003700	Transcription factor activity, sequence‐specific DNA binding	15	3.92 × 10^−5^	CEBPA, JDP2, SOX6, ZEB1, SOX9, ZIC1, PROX1, SRF, MYCN, PGR, MEF2D, FOS, ETS1, NEUROD1, TCF4
GO: 0003677	DNA binding	20	4.29 × 10^−5^	CEBPA, KLF6, ZMYM2, EZH2, ARID1A, SOX6, ZEB1, TET2, ARID2, LIN28B, PROX1, MYCN, PGR, MAPK1, FOS, SP2, ETS1, ASH1L, TOP2B, TCF4
GO: 0030332	Cyclin binding	4	7.72 × 10^−5^	CUL3, FBXW7, PTCH1, CDK6
GO: 0046982	Protein heterodimerization activity	10	1.38 × 10^−4^	CUL3, MEF2D, FOS, CLCN3, JDP2, NEUROD1, SOX6, TOP2B, TCF4, SOX9
GO: 0003682	Chromatin binding	9	2.29 × 10^−4^	FOS, JDP2, EZH2, ASH1L, NEUROD1, ZEB1, TOP2B, TCF4, SOX9
GO: 0005102	Receptor binding	8	6.95 × 10^−4^	PGR, FGA, CADM2, GJA1, JAK2, ABCA1, CXCL12, CDH5

**Table 5:** GO functional annotation for most significantly related targets of miR‐101‐5p

GO ID	Term	Gene no.	*P*	Genes
BP				
GO: 0043066	Negative regulation of apoptotic process	29	2.01 × 10^−13^	YWHAZ, MTDH, ERBB4, XIAP, IL6ST, FOXO1, PRKDC, RPS6KB1, IGF1R, DDX3X, BCL2, TPT1, GLO1, MYC, TWIST1, BMP4, IL6, TBX3, SOCS2, SMAD3, *et al*.
GO: 0045893	Positive regulation of transcription, DNA‐templated	28	2.34 × 10^−11^	RSF1, ERBB4, FOXO1, ZIC1, ASPH, NFATC2, MYC, BMP4, KLF6, IL6, TBX3, TGFBR1, CREBBP, SMAD5, SMAD4, ESR1, ATAD2, SMAD3, SMAD2, ESR2, *et al*.
GO: 0045944	Positive regulation of transcription from RNA polymerase II promoter	38	5.43 × 10^−11^	PRKDC, FOXO1, ZEB2, NR3C1, SOX6, ZEB1, ZIC1, PGR, IL17A, BARX2, CDKN2B, DDX3X, ZNF148, NFATC2, YES1, MYC, TWIST1, CKAP2, BMP4, IL6,*et al*.
GO: 0000122	Negative regulation of transcription from RNA polymerase II promoter	32	9.99 × 10^−11^	JDP2, MTDH, USP2, FOXO1, ZEB2, SOX6, ZEB1, BARX2, ZNF148, NFATC2, MYC, TWIST1, BMP4, DAB2IP, TBX3, YY1, CREBBP, SMAD4, ESR1, KLF17, *et al*.
GO: 0008284	Positive regulation of cell proliferation	25	4.77 × 10^−10^	ERBB4, IL6ST, IGF1R, CD47, KRAS, TNFRSF11A, ITGAV, BCL2, MYC, IL6, FLT1, KLB, TBX3, TGFBR1, PROX1, TET1, LEP, MAPK1, HDAC2, CRKL, *et al*.
GO: 0043065	Positive regulation of apoptotic process	19	7.80 × 10^−9^	BMP4, IL6, DAB2IP, ERBB4, PTGS2, PRKDC, FOXO1, FRZB, LATS1, BCL2L11, ATM, BAK1, TRIM35, ITGA6, DDX3X, SFRP1, ATG7, SOS1, UNC5C
GO: 0050900	Leukocyte migration	12	1.23 × 10^−7^	NRAS, CD47, KRAS, ITGA6, ITGAV, SOS1, TREM1, YES1, ANGPT2, MMP1, SLC7A11, PTPN11
GO: 0008285	Negative regulation of cell proliferation	18	2.36 × 10^−6^	BMP4, IL6, DAB2IP, ERBB4, PTGS2, SMAD4, SMAD2, CDK6, ZEB1, FRZB, ARID2, PROX1, SLIT3, BAK1, SPRY1, CDKN2B, SFRP1, MDM4
GO: 0001568	Blood vessel development	7	3.73 × 10^−6^	MIB1, LAMA4, CRKL, TBX3, ITGAV, FOXO1, AHR
GO: 0071498	Cellular response to fluid shear stress	5	7.02 × 10^−6^	MTSS1, PTGS2, CA2, NFE2L2, TFPI2
CC				
GO: 0005829	Cytosol	67	3.59 × 10^−8^	RPL36A, FOXO1, RPS6KB1, LATS1, MAP3K7, CCNE2, BAK1, SPRY1, GSTM3, CDKN2B, MAT1A, ATG7, MYC, PRKCA, DAB2IP, SOCS2, SGK3, RALBP1, G3BP1, CYCS, *et al*.
GO: 0005654	Nucleoplasm	53	1.23 × 10^−5^	RSF1, XPO4, FOXO1, RPS6KB1, ZEB1, ZIC1, PGR, CCNE2, SPRY1, CDKN2B, ZNF148, MYC, AKT3, PRKCA, DTL, ESR1, CDK6, ESR2, AHR, MCM6, *et al*.
GO: 0009897	External side of plasma membrane	12	1.50 × 10^−5^	EPHA5, VCAM1, CLCN3, IL17A, IL6, TNFRSF11A, ITGA6, CD40LG, IL6ST, ITGAV, CD274, IL7R
GO: 0005634	Nucleus	84	2.85 × 10^−5^	JDP2, RSF1, PTGS2, CPEB4, FOXO1, ZEB2, RPS6KB1, ZEB1, ZIC1, MAP3K7, CCNE2, PGR, GSTM3, BARX2, CDKN2B, TPT1, LOX, TFPI2, MYC, ANGPT2, *et al*.
GO: 0005737	Cytoplasm	81	4.65 × 10^−5^	MTSS1, PTGS2, XPO4, CPEB4, FOXO1, RPS6KB1, ZEB1, ZIC1, MAP3K7, SPRY1, GSTM3, CDKN2B, ATG7, TPT1, FRS2, AKT3, PRKCA, DAB2IP, SOCS2, LPGAT1, *et al*.
GO: 0071141	SMAD protein complex	4	6.01 × 10^−5^	SMAD5, SMAD4, SMAD3, SMAD2
GO: 0005667	Transcription factor complex	10	1.97 × 10^−4^	BARX2, YY1, SMAD5, SMAD4, SMAD3, PRKDC, SMAD2, DACH1, ZEB1, AHR
GO: 0043235	Receptor complex	8	3.74 × 10^−4^	IGF1R, FLT1, ERBB4, LEPR, TGFBR1, NTRK2, SMAD3, GHR
GO: 0009986	Cell surface	16	5.76 × 10^−4^	CLCN3, TGFBR1, MET, RPS6KB1, CFTR, SDC2, SLC7A11, VCAM1, ITGA6, SULF2, PRLR, SFRP1, CD40LG, ITGAV, CNTN2, GHR
GO: 0005622	Intracellular	27	1.45 × 10^−3^	PRKCA, KLB, TGFBR1, G3BP1, SMAD5, SOCS6, SMAD4, DCDC2, SMAD3, MAPK10, LATS1, SEC63, WSB1, MAPK1, RND3, NRAS, TRIM35, KRAS, SFRP1, TRIM33, *et al*.
MF				
GO: 0005515	Protein binding	147	5.59 × 10^−14^	MTSS1, RPL36A, JDP2, PTGS2, IL6ST, XPO4, FOXO1, RPS6KB1, PGR, MAP3K7, CD47, BAK1, CDKN2B, ATG7, TPT1, ASPH, LOX, FRS2, TWIST1, DAB2IP, *et al*.
GO: 0008134	Transcription factor binding	18	1.69 × 10^−8^	YWHAZ, CREBBP, ESR1, SMAD3, PRKDC, SMAD2, ZEB1, AHR, MAPK1, HDAC2, SP1, DDX3X, PSMD10, ATG7, BCL2, NFATC2, MYC, TWIST1
GO: 0001078	Transcriptional repressor activity, RNA polymerase II core promoter proximal region sequence‐specific binding	10	3.79 × 10^−6^	JDP2, TBX3, ZNF148, YY1, CREBBP, KLF17, FOXO1, DACH1, NFATC2, PROX1
GO: 0005524	ATP binding	37	5.87 × 10^−6^	CLCN3, ERBB4, PFKFB2, STK17B, PRKDC, RPS6KB1, LATS1, MAP3K7, IGF1R, KRAS, DDX3X, MAT1A, HSPE1, YES1, AKT3, PRKCA, FLT1, SGK3, TGFBR1, UBE4B, *et al*.
GO: 0000978	RNA polymerase II core promoter proximal region sequence‐specific DNA binding	15	4.05 × 10^−5^	JDP2, TBX3, SMAD4, ESR1, KLF17, SMAD3, SMAD2, NR3C1, ZIC1, PGR, HDAC2, SP1, ZNF148, NFATC2, MYC
GO: 0042802	Identical protein binding	22	8.15 × 10^−5^	MTSS1, YWHAZ, DAB2IP, XIAP, USP2, SMAD4, ESR1, SMAD3, CLDN10, RPS6KB1, STAT2, MCM6, PBLD, IGF1R, BAK1, MAPK1, GLUL, GSTM3, SFRP1, BCL2, CNTN2, GBP1
GO: 0008270	Zinc ion binding	29	8.19 × 10^−5^	RSF1, XIAP, NR3C1, ZEB1, LIN28B, MMP1, PGR, GLO1, XAF1, RCHY1, PRKCA, ZMYM2, YY1, CREBBP, ESR1, SMAD3, WHSC1, ESR2, TET2, TET1, *et al*.
GO: 0004672	Protein kinase activity	14	1.79 × 10^−4^	PRKCA, SGK3, TGFBR1, MET, MAP2K4, STK17B, PRKDC, RPS6KB1, PBK, MAPK10, MAP3K7, MAP4K4, HIPK2, AKT3
GO: 0043565	Sequence‐specific DNA binding	17	2.04 × 10^−4^	JDP2, TBX3, SMAD4, ESR1, SMAD3, FOXO1, WHSC1, NR3C1, ESR2, SOX6, PGR, HDAC2, SP1, ZNF148, BCL2, NFE2L2, MYC
GO: 0019899	Enzyme binding	13	3.32 × 10^−4^	PRKCA, PTGS2, UBE4B, ESR1, PRKDC, CFTR, ESR2, PGR, GSTM3, HDAC2, MDM4, YES1, GBP1

#### Protein–protein interaction network

**Figure 12:**
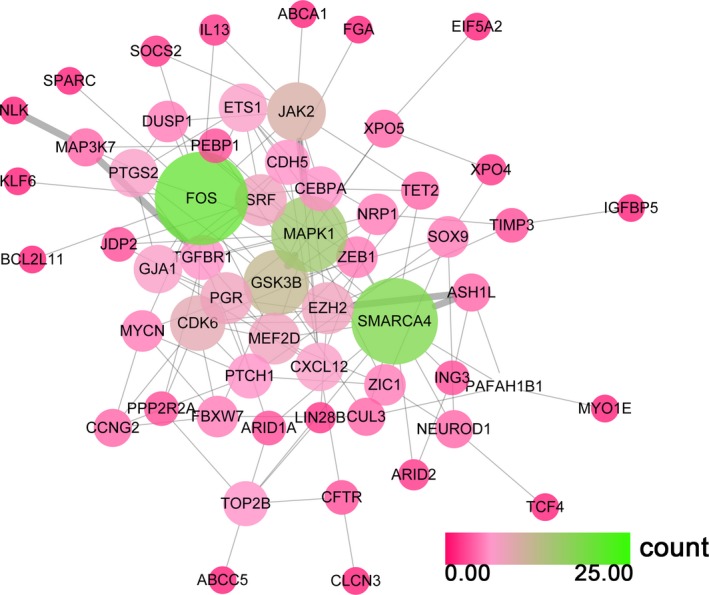
The PPI network of miR‐101‐3p potential targets. Both the color and the size of the nodes reflect the connectivity degrees of two nodes; nodes with a green color are perceived as hub genes.

**Figure 13:**
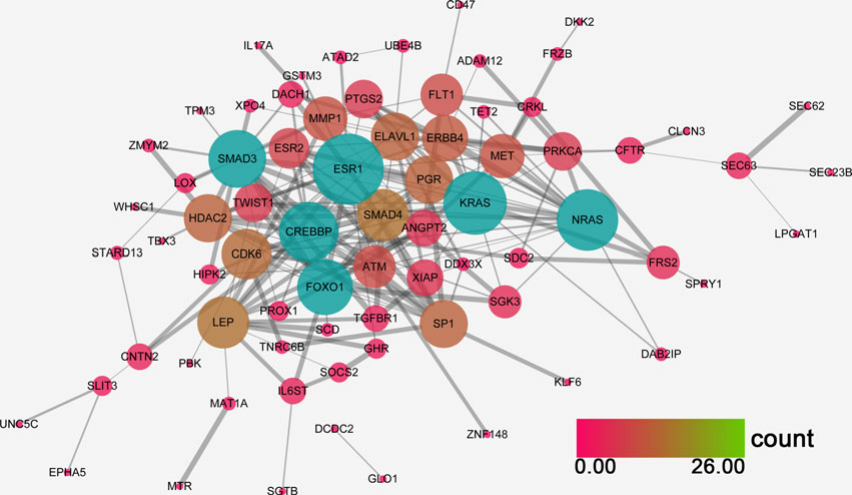
The PPI network of miR‐101‐5p potential targets. Both the color and the size of the nodes reflect the connectivity degrees of two nodes; nodes with a blue color are perceived as hub nodes.

A PPI network was designed to screen out the hub genes according to the degree to which each of the genes appeared in the network. Here, the PPI network was constructed by using the STRING database. As shown in Figs 12 and 13, *FOX*,* SMARCA4* and *MAPK1* remained the top three utmost important genes for miR‐101‐3p, while *ESR1*,* KRAS*,* NRAS*,* FOXO1*,* CREBBP* and *SMAD3* were regarded as the hub genes for miR‐101‐5p.

## Discussion

In the present study, we investigated the relationship between miR‐101 expression and clinicopathological parameters. TCGA data showed that the miR‐101 level was significantly lower in HCC than in para‐non‐cancerous liver tissues, and great diagnostic value of miR‐101 in HCC was found. Additionally, miR‐101 was negatively correlated with pathological stage, pathological T stage and histological grade. Since these clinicopathological parameters are indicators of tumor deterioration and progression, monitoring the level of miR‐101 may have a certain significance in the progression of HCC.

Accumulating studies have indicated that dysregulation of circulating miRNAs could be a biomarker of tumorigenesis, development and invasion in various cancers including prostate cancer, gastric cancer, ovarian cancer, breast cancer and lung cancer 30, 46, 47, 48, 49. A diagnostic value for circulating miR‐101‐3p/5p in HCC has also been reported 50, 51. Both of these studies validated that a lower miR‐101‐3p/5p level had diagnostic potential for HCC. However, due to the limited number of available publications, the exact diagnostic value of miR‐101 and the difference between miR‐101‐3p and miR‐101‐5p are still unclear. Alpha‐fetoprotein (AFP), as the traditional marker of liver diseases, has been used for HCC diagnosis in the clinic. Recently, He *et al*. 33 conducted a meta‐analysis with 10 data sets (879 HCC patients and 1028 controls) assessing AFP for HCC diagnosis and revealed that the AUC–SROC of pooled AFP was 0.82 (95% CI: 0.78–0.85), with sensitivity of 0.631 (95% CI: 0.552–0.703) and specificity of 0.943 (95% CI: 0.875–0.975). Here, we first combined gene expression microarray datasets from the GEO database and RNA‐seq from TCGA database, as well as two studies, to further confirm the diagnostic efficacy of miR‐101‐3p and miR‐101‐5p and then discover the difference between the two mature mRNAs. Our findings suggested that the pooled diagnostic accuracy of miR‐101‐3p for HCC (SROC: 0.86 (95% CI: 0.82–0.89); sensitivity and specificity were 78.0% (95% CI: 65.0–88.0%) and 79.0% (95% CI: 0.67–88.0%), respectively), which showed a slightly higher diagnostic value than AFP. As for miR‐101‐5p, the SROC was 0.80 (95% CI: 0.76–0.83), a little bit lower than AFP, but it also showed a moderate value for HCC diagnosis, which is comparable to AFP's diagnostic value.

Even though miR‐101‐3p/5p expression showed a high diagnostic value for HCC, the heterogeneity among the studies must be considered. Since our study indicated that heterogeneity from the threshold effect was absent, we deduced that the heterogeneity may be caused by the different data platforms and the large gaps between each study. Considering that the number of studies was small, we did not conduct a subgroup analysis.

Subsequently, bioinformatic analysis was performed to determine the molecular mechanism of miR‐101‐3p/5p in HCC. In the past, researchers exploring the molecular mechanism of miRNAs only concentrated on one or two target genes. For example, Varambally *et al*. 52 first reported that *EZH2* was the target gene of miR‐101 (3p and 5p were not distinguished) several years ago. Another study confirmed that miR‐101 (3p and 5p were not distinguished) inhibits HCC progression and metastasis through *EZH2* down‐regulation 53. Liu *et al*. 54 identified another target gene of miR 101 (3p and 5p were not distinguished), *VEGF C*, which promotes invasion and migration. *MCL‐1* and *COX‐2*, which play a role in tumorigenesis, have also been identified as the target genes of miR‐101 (3p and 5p were not distinguished) 31, 55. In addition, metastasis of HCC has been shown to be affected by different target genes of miR‐101 (3p and 5p were not distinguished), such as *STMN1* 56 and *PTEN* 57. Since a single miRNA can target multiple genes to achieve its biological and clinical functions, the exploration of the relevant gene network can reveal the widespread molecular mechanism of miR‐101‐3p/5p. Hence, we identified potential target genes of miR‐101‐3p/5p *in silico*. Moreover, we further narrowed the list by analyzing the genes that overlapped with the differentially expressed genes of HCC identified via NLP. Next, these target genes were subjected to KEGG pathway annotation and GO enrichment analysis by using the DAVID. The target genes of both miR‐101‐3p and miR‐101‐5p are involved in pathways in cancer, hepatitis B and the MAPK signaling pathway. These results reveal that miR‐101 probably contributes to the tumorigenesis and metastasis of HCC. Previous studies have reported the role of these pathways in liver cancer 58, 59. The GO term analysis indicated that these potential target genes of miR‐101‐3p/5p were significantly involved in the regulation of the cell cycle and cell proliferation, which are associated with tumor occurrence or stepwise development.

Furthermore, we constructed the PPI network with potential target genes, showing that miR‐101‐3p probably targets *FOS*,* SMARCA4*,* MAPK1*,* GSK3B* and *JAK2* to exert its function in HCC. Li *et al*. 60 reported that *FOS* acts as a regulator of cell proliferation, differentiation and transformation, and miR‐101 inhibits cell invasion and migration via down‐regulation of *FOS*. MAPK1 has also been reported to be involved in a variety of cellular processes, such as differentiation, proliferation and development through the MAPK pathway 61. JAK2 is a protein tyrosine kinase, and recent evidence has demonstrated that miR‐101 (3p and 5p were not distinguished) inhibits breast cancer cell proliferation and promotes apoptosis by targeting JAK2 49. Previous studies reveal that miR‐101‐3p might regulate the occurrence and development of HCC by targeting various genes, and tumorigenesis probably results from the abnormality of multiple genes. Of course, the correlation of those potential key genes of miR‐101‐3p needs further experimental validation. Next, a functional analysis of these target genes *in vitro* and *in vivo* will need to be conducted, such as by RNA interference and cellular transfection, luciferase reporter assay, western blot and so on. miR‐101‐5p possibly targets *ESR1*,* KRAS*,* CREBBP*,* FOXO1* and *SMAD3* through different pathways. Among them, *KRAS*, a Kirsten ras oncogene homolog, was reported to have functional synergy with HBx in HCC initiation and progression 62, and *FOXO1* has been proposed to inhibit EMT transcriptional activators in HCC 63, 64. Additionally, Hishida *et al*. 65 indicated that *ESR1* is a tumor suppressor gene in HCC. Taken together, the hub genes identified may perform key roles in HCC. Further investigation appears to be necessary to confirm their exact function in HCC.

Taken together, the present study validated the down‐regulation of the two opposing strands, miR‐101‐3p and miR‐101‐5p, in HCC clinical specimens; however, miR‐101‐3p held a greater value for HCC diagnosis. Bioinformatic analysis revealed that miR‐101‐3p and miR‐101‐5p are involved in the same or similar signaling pathways through regulating a different set of target genes. The fact that miR‐101‐3p and miR‐101‐5p are involved in these signaling pathways suggests that the expression of miR‐101‐3p and miR‐101‐5p is close in HCC tissues, and they may function cooperatively with each other in the differentiation, proliferation and development of HCC.

In conclusion, we provide a comprehensive analysis of miR‐101‐3p/5p and evaluated the value of miR‐101‐3p and miR‐101‐5p as biomarkers for the early diagnosis of HCC. In addition, we investigated the prospective molecular mechanisms of these two opposing strands *in silico*. Our results provide a deeper understanding of the role of miR‐101‐3p/5p in HCC and facilitate the possible development of a miRNA‐based targeted therapy of HCC. However, several limitations should be considered in this study. First, the total number of studies included was limited; second, further experiments *in vitro* and *in vivo* are still required to confirm the function of the target genes.

## Author contributions

XY and Y‐YP contributed equally in this study. XY, Y‐YP and PL collected data and wrote the paper draft; R‐QH and J‐MC contributed to the analysis; JM, GC and HY designed the study, supervised the report and corrected the manuscript.
